# *Pfi*t Is a Structurally Novel Crohn's Disease-Associated Superantigen

**DOI:** 10.1371/journal.ppat.1003837

**Published:** 2013-12-26

**Authors:** Lihui Liu, Hui Chen, Matthew B. Brecher, Zhong Li, Bo Wei, Bisweswar Nandi, Jing Zhang, Hua Ling, Gary Winslow, Jonathan Braun, Hongmin Li

**Affiliations:** 1 Wadsworth Center, New York State Department of Health, Albany, New York, United States of America; 2 Department of Pathology and Laboratory Medicine, David Geffen School of Medicine, University of California, Los Angeles, Los Angeles, California, United States of America; 3 Department of Biomedical Sciences, School of Public Health, State University of New York, Albany, New York, United States of America; Washington University, United States of America

## Abstract

T cell responses to enteric bacteria are important in inflammatory bowel disease. I2, encoded by the *pfi*T gene of *Pseudomonas fluorescens*, is a T-cell superantigen associated with human Crohn's disease. Here we report the crystal structure of *pfi*T at 1.7Å resolution and provide a functional analysis of the interaction of *pfi*T and its homolog, PA2885, with human class II MHC. Both *pfi*T and PA2885 bound to mammalian cells and stimulated the proliferation of human lymphocytes. This binding was greatly inhibited by anti-class II MHC HLA-DR antibodies, and to a lesser extent, by anti HLA-DQ and DP antibodies, indicating that the binding was class II MHC-specific. GST-*pfi*T efficiently precipitated both endogenous and *in vitro* purified recombinant HLA-DR1 molecules, indicating that *pfi*T directly interacted with HLA-DR1. Competition studies revealed that *pfi*T and the superantigen *Mycoplasma arthritidis* mitogen (MAM) competed for binding to HLA-DR, indicating that their binding sites overlap. Structural analyses established that *pfi*T belongs to the TetR-family of DNA-binding transcription regulators. The distinct structure of *pfi*T indicates that it represents a new family of T cell superantigens.

## Author Summary

Human inflammatory bowel disease (IBD) is a family of chronic inflammatory disorders of the gastrointestinal tract which affect genetically susceptible individuals. IBD is a lifelong disease involving mostly young people, often severely. Crohn's disease (CD) and ulcerative colitis are the two major forms of IBD. Although the exact cause of these diseases remains unknown, both genetic and environmental factors together play significant roles in the disease pathogenesis. Several lines of evidence implicate commensal bacteria as an important pathogenic element in clinical disease, particularly in CD. We recently identified a novel microbial gene, I2, encoded by *Pseudomonas fluorescens*, a gram-negative commensal, which may be involved in the pathogenesis of CD. Both molecular and immunological approaches were used to identify the human receptor for the microbial antigen encoded by I2, to characterize the ligand-receptor interactions, and to determine the three-dimensional structure of the microbial gene product. In particular, we show that the *pfi*T is a T cell superantigen, which may help to explain how microbial flora can trigger immune activation in IBD, and may provide the groundwork for novel therapies to treat CD.

## Introduction

T-cell receptors (TCRs) typically recognize antigens in the form of peptides or peptide-lipids bound to major histocompatibility complex (MHC) molecules. TCRs and class II MHC proteins may directly interact with viral or bacterial proteins known as superantigens (SAgs) to activate T cells. Strong primary T cell responses are elicited by microbial toxin superantigens *via* their interaction with TCR Vβ [1], [2], [3], [4], [5] elements, and in some cases to TCR Vα chains [6], [7], [8].

A novel enteric T cell superantigen, I2, and its full-length gene product *pfi*T, are encoded by a microbial gene associated with Crohn's disease (CD) [9], [10], [11]. Several lines of evidence has implicated I2 and *pfi*T in the pathogenesis of CD [10], [12], [13], [14], [15], [16], [17], [18]. The I2 sequence was selectively detected in active CD lesional colonic tissue, but not in healthy controls or inactive CD ileum. Serum antibody studies revealed that IgA seroreactivity to a recombinant I2 protein was CD specific. Such IgA antibodies specific for I2 were detected in 50–60% of patients (both children and adults) with CD, 10% of patients with ulcerative colitis, and 4% of healthy controls. Patients expressing I2 were distinguished by greater disease severity and progression, including a greater frequency of strictures, internal perforations, and small bowel surgery (72% in antibody-positive patients, versus 23% in antibody-negative patients) [19].

Through genetic analysis and genomic cloning, a full-length I2 gene (*pfi*T) was identified as a genomic element of *Pseudomonas fluorescens*, a normal environmental bacterium detectable in the human gastrointestinal tract [11]. Commensal bacteria have emerged as an important factor in the pathogenesis of CD [20], although *P. fluorescens* has had limited testing as a potential pathobiont in IBD [21].

Several lines of evidence suggest that the I2 protein *pfi*T is a novel T-cell SAg [9], [11]. I2 protein induced proliferation and IL-10 responses by normal CD4^+^ T cells [9]. The I2 response was dependent on MHC class II-mediated recognition, but the antigen did not require antigen processing, a feature typical of SAgs, and antibody blockade of I-A^b^ specifically blocked the T-cell proliferative response [9]. Selective activation was observed for the murine TCR-Vβ5 subpopulation, and *pfi*T also exhibited T-cell SAg bioactivity [11]. PA2885, the apparent *Pseudomonas aeruginosa* homolog of *pfi*T (78% sequence identity) also stimulated murine CD4^+^ T cell proliferation [11]. Accordingly, colonization by the I2 and *pfi*T-expressing microorganism, *P. fluorescens*, in IBD susceptible hosts, was speculated to provide a superantigenic stimulus pertinent to the pathogenesis of Crohn's disease.

In this study, we describe structural and functional studies of the CD-associated superantigen *pfi*T. We show that *pfi*T can stimulate the activation of both mouse splenocytes and human peripheral blood mononuclear cells (PBMCs). We show that *pfi*T specifically and directly interacts with class II MHC HLA-DR, and to a lesser extent HLA-DP or HLA-DQ, and show that *pfi*T shares an overlapping binding site on HLA-DR1 with other superantigens. Crystal structure of *pfi*T at high resolution revealed that *pfi*T belongs to the bacterial transcription factor family of tetracycline repressor (TetR). The distinct structure of *pfi*T relative to other SAgs suggests that it represents a novel family of SAg.

## Results

### Expression and purification of full-length *pfi*T and PA2885

**Figure 1:**
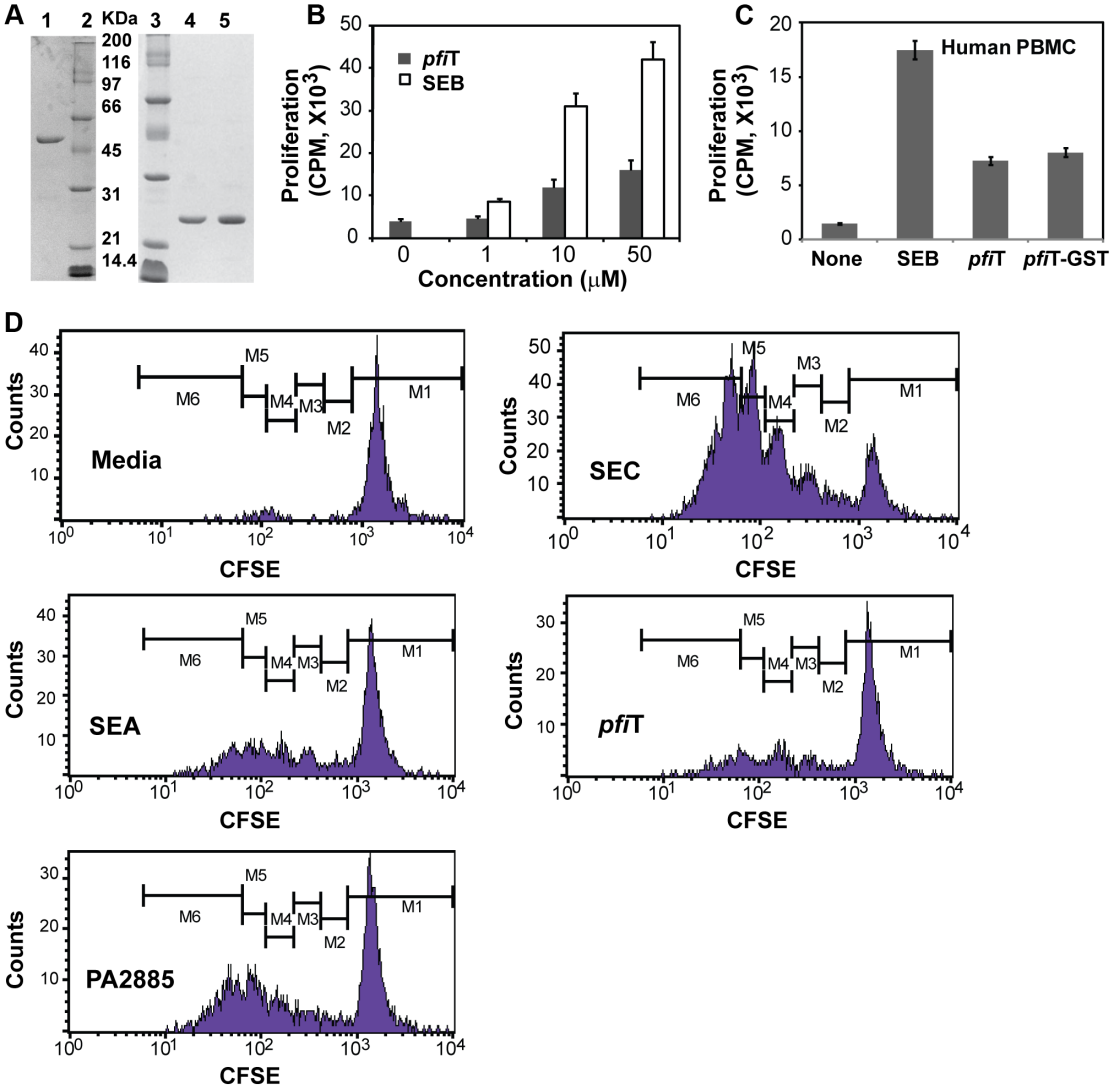
Soluble recombinant *pfi*T and PA2885 stimulate the activation of lymphocytes. (**A**) SDS-PAGE analysis of purified recombinant *pfi*T and PA2885 proteins. Lane 1, purified *pfi*T-GST fusion protein; Lanes 2 and 3, molecular weight standard; Lane 4, purified *pfi*T; Lane 5, purified PA2885. (**B**) Stimulation of murine splenocytes by *pfi*T and control SAg SEB. (**C**) Stimulation of human PBMC by *pfi*T, *pfi*T-GST fusion protein, and control SAg SEB. (**D**) Proliferation profiles of human T cells labeled with CFSE, stimulated by *pfi*T, PA2885, and control SAgs.

Attempts to express and purify soluble His-tag versions of I2, *pfi*T, and PA2885 failed. Therefore, we grafted the inserts into the pGEX-6P-1 vector (GE HealthCare) and expressed the proteins as GST-fusion proteins. Although GST-I2 remained insoluble, GST-*pfi*T and GST-PA2885 could be directly affinity-purified from cell lysates under native conditions (**Fig. 1A**). To obtain tag-less proteins, the GST-fusion proteins were digested by the PreScission protease (GE HealthCare). All proteins, tagged or untagged, were further purified to homogeneity using size-exclusion chromatography (**Fig. 1A**).

### *pfi*T stimulates the activation of both mouse splenocytes and human PBMC

Previously, I2 and *pfi*T were found to stimulate the activation of murine T cells with features most representative of a T cell superantigen [9], [11]. However, the proteins used in previous studies were purified from bacterial inclusion bodies and were possibly non-native. Therefore, we evaluated whether the proteins purified from soluble fractions were also immunostimulatory. Using *pfi*T purified directly from soluble fractions, activation of murine T cells was detected in a dose-dependent manner, although the magnitude of stimulation by *pfi*T was not as strong as that induced by the most potent known superantigen Staphylococcal enterotoxin (SE) B (SEB) (**Fig. 1B**).

Since the I2 gene was isolated from human CD patients, we anticipated that *pfi*T, the full-length protein encoded by I2, could act on human T lymphocytes. Therefore, we purified human T cells from peripheral blood mononuclear cells (PBMCs), and assayed T-cell proliferation in response to *pfi*T. As shown in **Fig. 1C**, both recombinant *pfi*T and its GST-fusion protein activated human T lymphocyte proliferation. To verify that *pfi*T and PA2885 could both activate human T cells, we labeled purified human T lymphocytes with the fluorescent dye carboxyfluorescein succinimidyl ester (CFSE) that permits visualization of proliferation by dye dilution during successive rounds of cell division. CFSE-labeled human T lymphocytes were then cultured with *pfi*T or PA2885; SEC and SEA were used as positive control SAgs, and media alone was used as a negative control. FACS analysis confirmed that CFSE-labeled human T-cells did not respond to the media control (**Fig. 1D**), but proliferated robustly to the SAg SEC (up to 5 generational CFSE-decremented populations, **Fig. 1D**). Although not as robust as SEC, *pfi*T and PA2885 triggered substantial T cell proliferation, comparable to levels induced by another positive control SAg, SEA (**Fig. 1F–H**). Overall, our results indicated that recombinant soluble *pfi*T and PA2885 were biologically active and could induce proliferation of both murine and human T cells.

### Soluble *pfi*T binds to the cell surface of lymphocytes

**Figure 2:**
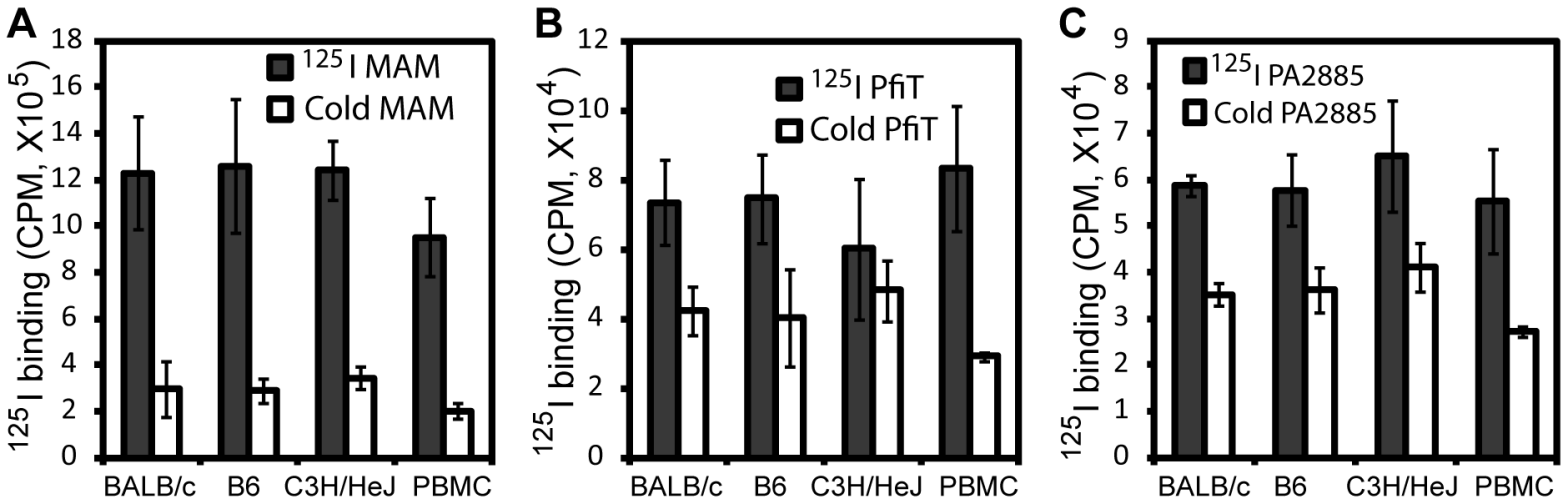
Analysis of binding of ^125^I-lebdeled superantigens to murine splenocytes and human PBMC. (**A**) MAM (control SAg); (**B**) *pfi*T; (**C**) PA2885. Experiments were performed in triplicate in the presence or absence of “cold” protein competitors.

In order to identify human receptors responsible for presentation of *pfi*T and PA2885, we first performed an ^125^I-protein competition assay to investigate whether recombinant *pfi*T and PA2885 could bind lymphocytes. As test proteins, we labeled *pfi*T and PA2885 with ^125^I; as a positive control SAg, the *Mycoplasma arthritidis*-derived mitogen (MAM) was also produced and labeled, as described previously [7], [22]. Unlabeled MAM, *pfi*T, and PA2885 were used as cold competitors in a competition binding assay, as described previously [22]. As expected, 10-fold molar excess of MAM greatly (>75%) diminished binding of ^125^I-labeled MAM to the cell surface of both murine splenocytes and human PBMC (**Fig. 2A**). Similarly, excessive quantities of *pfi*T and PA2885 reduced about 40% of the binding of the proteins to murine splenocytes. Specific binding was much lower in C3H/HeJ mice (only 20% competition), perhaps reflecting a lower avidity for the class II MHC proteins of this haplotype. These results indicated that *pfi*T and PA2885 specifically bind to mouse and human cells.

### Class II MHC is responsible for the binding of *pfi*T

**Figure 3:**
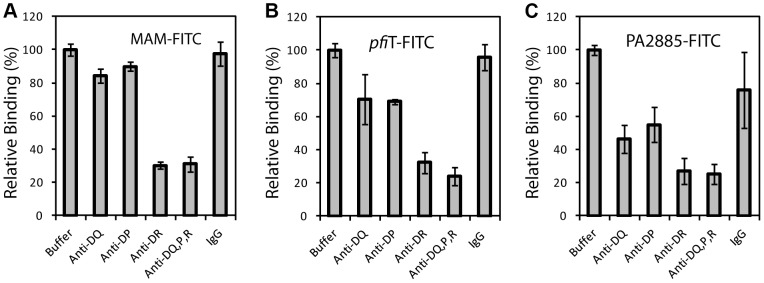
Binding of *pfi*T and PA2885 to human PBMC is dependent on the class II MHC HLA-DR. (**A**) Analysis of binding of FITC-labeled control SAg MAM to CD19^+^ human lymphocytes, in the presence of various anti-class II MHC and control antibodies. (**B**) Binding profile of FITC-labeled *pfi*T to CD19^+^ human lymphocytes. (**C**) Binding profile of FITC-labeled PA2885 to CD19^+^ human lymphocytes.

We anticipated that the binding of *pfi*T and PA2885 to PBMCs would be dependent on human class II MHC. To test this prediction, and specify the class II MHC proteins responsible for binding, we labeled *pfi*T, PA2885, and a control SAg MAM with fluorescein isothiocyanate (FITC). We then used flow cytometry to analyze the binding of FITC-labeled proteins to the surface of human PBMC in the presence of various blocking antibodies against class II HLA molecules. In control studies, antibodies targeting class II HLA-DR reduced the binding of FITC-labeled MAM to the surface of PBMC by 70% (**Fig. 3A**), whereas anti-HLA-DQ and anti-HLA-DP antibodies did not significantly affect the binding. This is consistent with the fact that MAM is presented primarily by HLA-DR [7], [23], [24]. When the binding of FITC-labeled *pfi*T to human PBMC was tested (**Fig. 3B**), we observed a significantly reduction (30% and 25% compared to buffer control) with an anti-HLA-DR antibody, or when using a polyclonal antibody recognizing all three class II alleles (DR, DQ, and DP). Anti-HLA-DQ and anti-HLA-DP antibodies also moderately decreased the binding of FITC-labeled *pfi*T to human PBMCs. For PA2885, all anti-HLA antibodies reduced binding by more than 45%, where the greatest inhibition was obtained using anti-HLA-DR (75%), or anti-DR, DQ, DP antibodies (**Fig. 3C**). Our results indicate that *pfi*T and PA2885 specifically bind to class II MHC, and primarily to HLA-DR.

**Figure 4:**
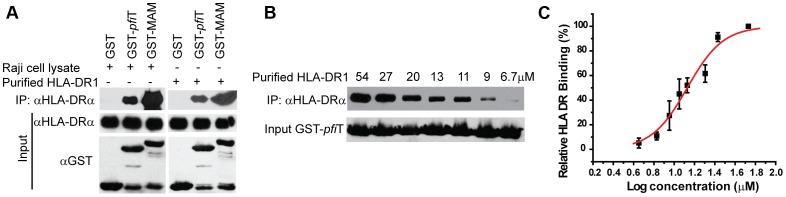
*Pfi*T directly interacts with the class II MHC HLA-DR. (**A**) Western blot analysis of interactions between *pfi*T and HLA-DR. *pfi*T-GST, control SAg GST-MAM, and negative control GST were used in GST pull down assay of the cell lysates of class II MHC HLA-DR+ Raji cells and purified recombinant HLA-DR expressed in *E. coli*. (**B**) *pfi*T binding to HLA-DR is dose dependent. Purified recombinant HLA-DR/HA complex was used. (**C**) Curve fitting to determine the affinity of *pfi*T binding to recombinant HLA-DR/HA complex.

To confirm these observations, we independently evaluated HLA binding using a GST immunoprecipitation assay (**Fig. 4**). GST, GST-*pfi*T and GST-MAM were expressed and purified to homogeneity, and were immobilized to glutathione affinity beads. Using a cell lysate of Raji cells as a source of endogenous HLA-DR molecules, GST-MAM and GST-*pfi*T fusion proteins, but not the free GST, precipitated HLA-DR molecules (**Fig. 4A**). These data provided additional evidence that GST-*pfi*T, like GST-MAM, interacted with HLA-DR molecules. Next, using a bacterial expression system, we produced, refolded, and purified recombinant HLA-DR1 protein bound to a hemagglutinin (HA) peptide, as described previously [24]. Using this recombinant HLA-DR1/HA complex, we performed the GST immunoprecipitation assay. Again, free GST failed to precipitate the recombinant complex, whereas both GST-*pfi*T and GST-MAM efficiently precipitated the HLA-DR1/HA complex. Since in the latter case, all the materials were highly purified recombinant proteins, the results indicated that *pfi*T directly interacts with the HLA-DR/HA complex. Moreover, data from both studies suggested that the binding of HLA-DR to GST-*pfi*T was less than to GST-MAM, indicating that *pfi*T binds HLA-DR with apparent lower affinity than MAM.

To determine the binding affinity of *pfi*T with HLA-DR, we used the GST immunoprecipitation assay to titrate the HLA DR/HA complex. As shown in **Fig. 4B and 4C**, binding of the recombinant HLA-DR/HA complex to GST-*pfi*T was dose-dependent. Non-linear regression of the titration curve revealed that the dissociation constant (*K_D_*) for *pfi*T-HLA-DR1/HA interaction was about 13.5 µM. By comparison, the *K_D_* value for MAM binding to HLA-DR1/HA was in the sub-nanomolar range [22], [25]. This result supports our observation that *pfi*T binds HLA-DR more weakly than does MAM.

### *pfi*T forms a dimer and binds HLA-DR1 in solution

**Figure 5:**
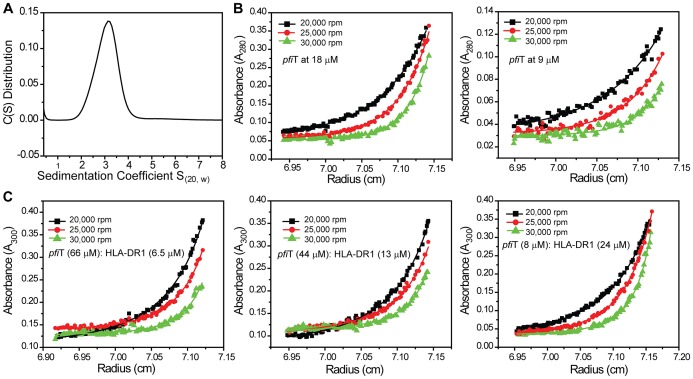
Analytical ultracentrifugation analysis of *pfi*T binding to recombinant HLA-DR1/HA complex. (**A**) Sedimentation coefficient distributions of *pfi*T alone at 3.6 µM concentration. (**B**) Sedimentation equilibrium analysis of *pfi*T. Representative absorbance distributions were shown for sedimentation equilibrium of *pfi*T at 18 µM (left panel) and 9 µM (right panel) at 20°C at rotor speeds of 20,000 rpm (black solid square), 25,000 rpm (red solid circle), and 30,000 rpm (green solid triangle). Distributions were analyzed as part of a global fitting to the absorbance data at multiple loading concentrations and multiple speeds. *Solid lines* are the global best-fit distributions using a reversible monomer and dimer model with SEDPHAT [27]. (**C**) Sedimentation equilibrium analysis of *pfi*T binding to the HLA-DR1/HA complex. Absorbance distributions were shown for sedimentation equilibrium of the *pfi*T-DR1 complex at different molar ratios at 20°C at rotor speeds of 20,000 rpm (black solid square), 25,000 rpm (red solid circle), and 30,000 rpm (green solid triangle). Data were analyzed as in (**B**).

To further investigate the interactions between *pfi*T and HLA-DR1, we performed analytical ultracentrifugation (AUC) sedimentation experiments. As it was known from our previous study [25] that HLA-DR1 exists as a monomer, we only performed sedimentation velocity (SV) experiment for *pfi*T to determine its oligomeration state. As shown in **Fig. 5A**, *pfi*T at a concentration of 3.6 µM showed a single peak with a sedimentation coefficient (S_(20, w)_) of 3.1 S. Transformation of C(S) distribution to c(M) size distribution [26] indicated a peak molecular weight (MW) of about 44 KDa, which is in a perfect agreement with a dimer form of *pfi*T with a MW of 45 KDa. Thus, our data demonstrate that *pfi*T forms a dimer in solution.

To determine the dissociation constant (*K_D_*) of *pfi*T dimer formation, we performed AUC sedimentation equilibrium experiments. By using SEDPHAT [27], we could readily model the sedimentation equilibrium data of *pfi*T with multiple concentrations and multiple speeds, using a single-species model. An apparent molecular mass of 48 KDa was obtained with a local root-mean-square (rms) deviation of 0.007 OD and a global χ^2^ of 1.5, with molecular mass being the only fitting parameter. This value is in good agreement with the theoretically calculated mass of 45 KDa for a *pfi*T dimer, suggesting again that *pfi*T exists as a dimer in solution. The somewhat high χ^2^ value indicated that a single-species model does not represent the experimental data very well. Indeed, when a monomer-dimer model was used in SEDPHAT [27], a much better fit was obtained, with a local rms deviation of 0.004 OD and a global χ^2^ of 0.7. A *K_D_* of 0.15 µM for the *pfi*T monomer-dimer equilibrium was obtained (**Fig. 5B**).

We next performed sedimentation equilibrium study of the *pfi*T/HLA-DR1 complex. *pfi*T was mixed with purified recombinant HLA-DR1/HA complex at three different molar ratios, and the sedimentation equilibrium was performed at three rotor speeds (**Fig. 5C**). Given that *pfi*T is a dimer and HLA-DR1 is a monomer, we chose a reversible (A+A)+B+B
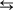
A+AB+B
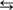
(AA)B+B
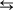
(AA)BB complex model, with A referring to *pfi*T, (AA) representing *pfi*T dimer, and B representing HLA-DR1. By using SEDPHAT [27], a global analysis of the equilibrium data gave an excellent fit, with a global reduced χ^2^ of 0.5 and local rms error of 0.004 OD (**Fig. 5C**). From this analysis, we estimated the binding affinity (*K_D_*(AB)) as 17 µM for *pfi*T binding to the HLA-DR1/HA complex. The binding affinity (17 µM) determined using the AUC sedimentation equilibrium is in agreement with the value of 13.5 µM determined using the GST pull-down assay, and is much lower than the affinity (0.1 µM) for MAM-binding to recombinant HLA-DR1 using the same AUC method [25]. Overall, our results indicate that recombinant proteins *pfi*T and HLA-DR1 bind each other in solution.

### The HLA-DR binding sites for *pfi*T and MAM overlap

**Figure 6:**
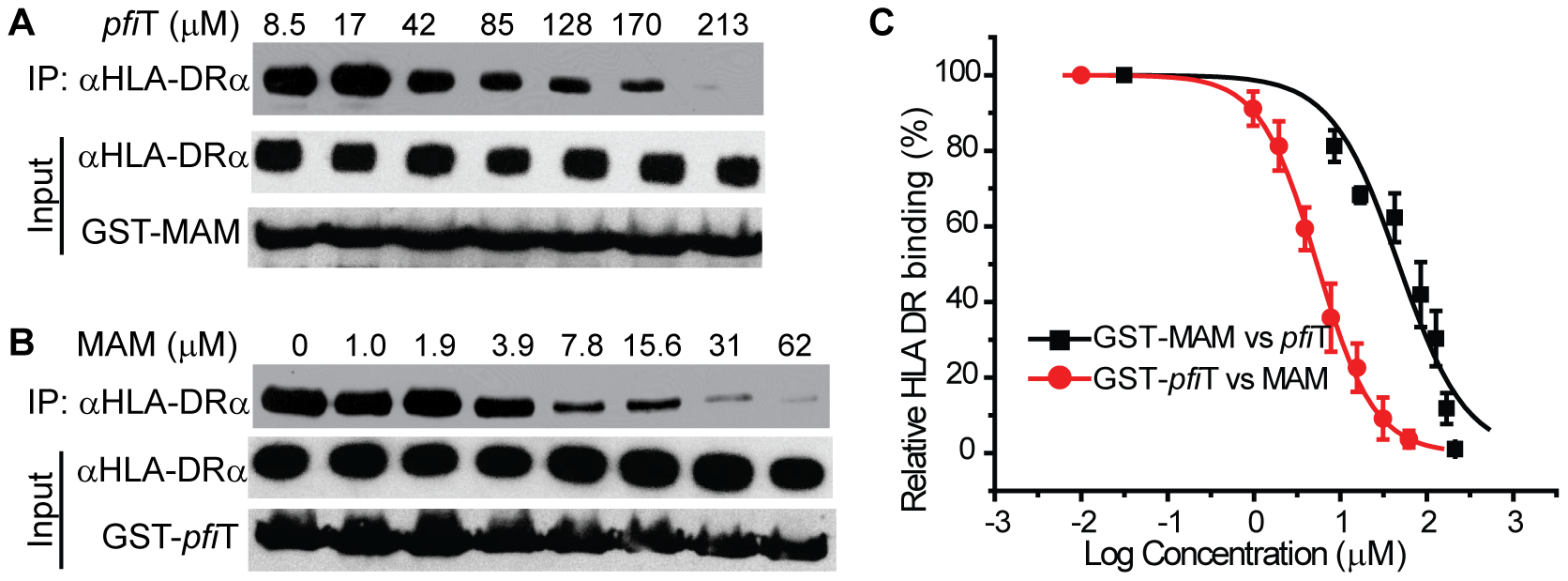
The binding site on HLA-DR for *pfi*T overlaps with that for MAM. (**A**) *pfi*T competes GST-MAM to bind HLA-DR in a dose-dependent manner. (**B**) MAM competes GST-*pfi*T to bind HLA-DR in dose-dependent manner. (**C**) Curve fitting to determine the IC_50_ (concentration required for competitors to inhibit 50% binding) for *pfi*T inhibition of MAM binding to HLA-DR or versus visa. Experiment was performed in duplicate.

In order to identify the site on HLA-DR to bind *pfi*T, we performed a competition GST immunoprecipitation assay, using tag-removed MAM or *pfi*T as a “cold” competitive binder. As shown in **Fig. 6A and 6C**, tag-less *pfi*T inhibited the binding of HLA-DR to immobilized GST-MAM in a dose-dependent manner; alternatively, free MAM decreased the binding of HLA-DR to GST-*pfi*T in a dose-responsive manner (**Fig. 6B,C**). Competition curve fitting of HLA-DR binding revealed that MAM inhibition of *pfi*T was more effective than *pfi*T inhibition of MAM. The *IC_50_* values for MAM inhibition of *pfi*T binding and *pfi*T inhibition of MAM binding to HLA-DR were 5.4 µM and 47 µM, respectively (*IC_50_*: inhibitor concentration required to inhibit ligand binding by 50%). These results were consistent with the GST immunoprecipitation assay (**Fig. 4A**), where a similar quantity of GST-*pfi*T precipitated less HLA-DR than did GST-MAM. Overall, these results indicate that the HLA-DR binding sites for *pfi*T and MAM overlap.

### Crystal structure of *pfi*T

**Figure 7:**
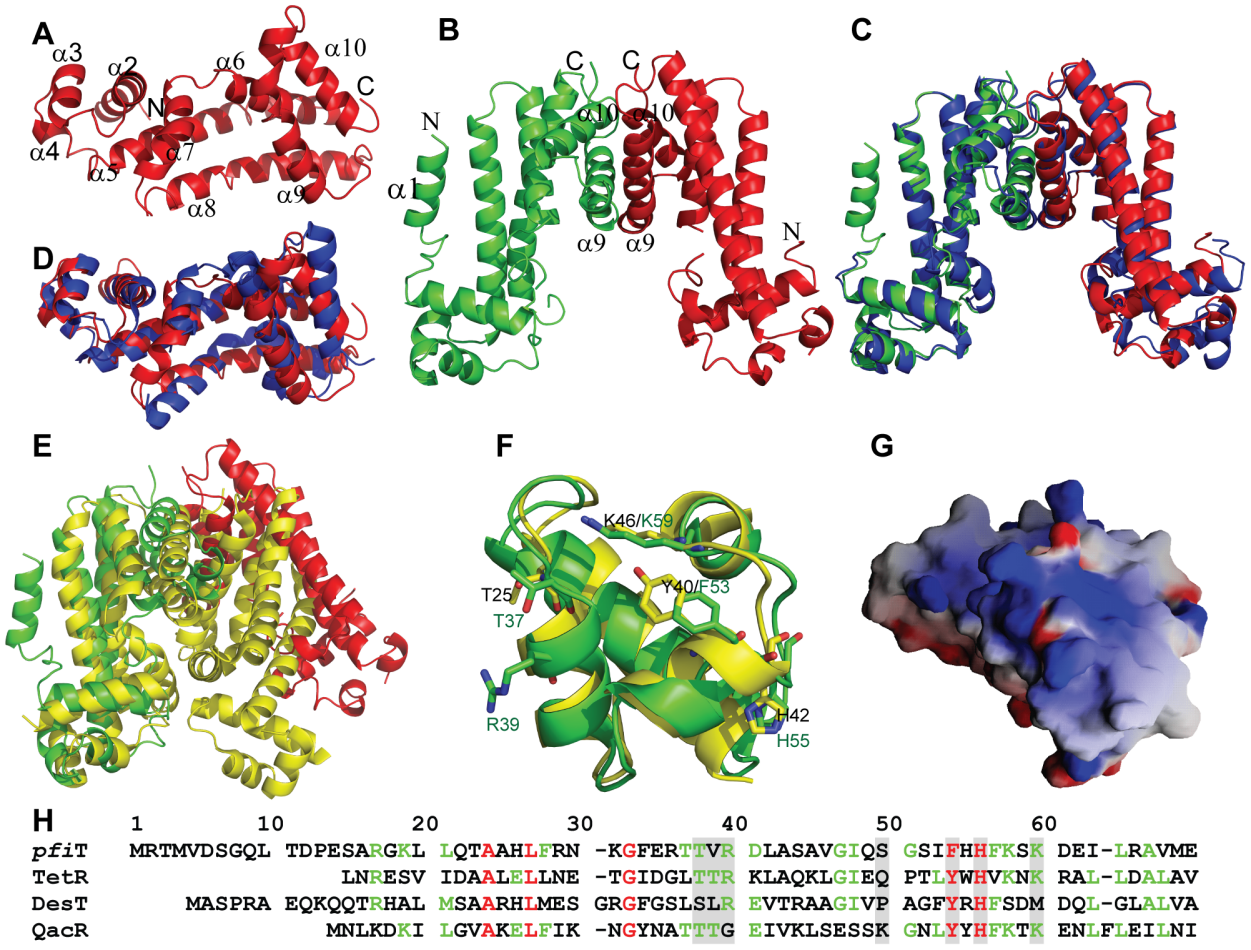
Crystal structure of *pfi*T. (**A**) Cartoon representation of crystal structure of native *pfi*T monomer. Secondary structural elements were labeled. (**B**) Cartoon representation of crystal structure of the *pfi*T dimer of the Se-Met *pfi*T crystal. The N-terminal α1 helix that is missing in the native *pfi*T structure and dimer interface helices were labeled. (**C**) Comparison of the dimer of Se-Met *pfi*T (green and red) with that of native *pfi*T (blue) reconstituted through crystallographic symmetry in the C2 crystal form. (**D**) Superposition of *pfi*T (red) to a putative TetR repressor (blue) from *Vibrio parahaemolyticus* (PDB code: 3HE0). (**E**) Superposition of *pfi*T dimer to that of the QacR-DNA complex (PDB: 1JT0) [37]. *pfi*T was colored as red and green for the two monomers of the dimer; the QacR dimer was colored yellow. (**F**) Structure comparison of *pfi*T with QacR at the QacR DNA-binding site. Residues that are important for DNA-binding were labeled in black (QacR) and green (*pfi*T), and presented in stick representation, with oxygen in red, nitrogen in blue, and carbon either in green (*pfi*T) or in yellow (QacR). (**G**) The electrostatic surface potential of *pfi*T at the putative DNA-binding site, with blue and red regions indicating positive and negative electrostatic regions, respectively. This figure was made with GRASP [57]. (**H**) Structure-based alignment of sequences of *pfi*T and TetR members (TetR [32], QacR [37], DesT [33]) with known three dimensional structures of protein-DNA complexes. Residues that make direct interactions with DNA elements were shaded. Residues were colored according to the extent of their sequence conservation: strictly conserved (red); >50% conservation (green); and not conserved (<50%) (black).

In order to better understand the biological function of this Crohn's disease-related SAg, we next determined the crystal structure of *pfi*T at 1.7 Å resolution (**Fig. 7** **,** **Table 1**). Native crystals were first grown with a space group C2 and one molecule per asymmetric unit. Since a sequence search indicated that *pfi*T belongs to the TetR-family transcription regulator [11], we attempted to solve the structure using molecular replacement (MR) method with various structures of the TetR members. However, none of the MR calculations gave clear solutions. We therefore generated seleno-methionine (Se-Met)-substituted *pfi*T protein. The Se-Met *pfi*T was crystallized in the *P*2_1_ space group with two molecules per asymmetric unit, which is different from that of the native crystal. After screening many crystals and diffraction data, we finally obtained clear structural solution at 2.4Å resolution from a set of diffraction data of the Se-Met *pfi*T crystal, using the single anomalous dispersion (SAD) phasing method. The structure was completed by iterative refinement and graphic modeling, using a set of diffraction data of the Se-Met crystal at 1.95Å resolution (**Table 1**). Structure of native crystals at 1.7Å resolution was determined using the MR method with the Se-Met *pfi*T structure as a search model (**Table 1**).

**Table 1:** Data collection, refinement, and model details.

	Native	Se-Met	Se-Met
***Data Collection***			
Space group	C2	P2_1_	P2_1_
Wavelength (Å)	1.0402	0.97923	0.97905
Cell parameters			
a (Å)	37.23	37.19	37.16
b (Å)	93.66	111.40	110.92
c (Å)	53.56	62.23	62.04
β (°)	109.82	100.28	100.15
Resolution range (Å)	25.2-1.7 (1.76-1.7)	45.7-2.4 (2.49-2.4)	45.7-1.95 (2.02-1.95)
No. of unique reflections	18843	19617	35150
Redundancy	3.0 (2.8)	4.8 (4.8)	3.6 (3.6)
Completeness (%)	99.6 (98.8)	99.9 (99.9)	97.1 (97.1)
Average *I/σ(I)*	20.3 (2.9)	29.1 (4.4)	30.6 (2.5)
*R_merge_* (%)	4.9 (55.6)	8.7 (46.7)	6.0 (69.4)
*R_ano_* (%)		7.2	
Se sites		13	
Figure of Merit		0.31	
***Refinement***			
Resolution limits (Å)	39.2-1.7		45.7-1.95
No. reflections	18840		35150
*R_work_* (%)	18.9		24.3
*R_free_* (%)	23.4		26.5
Non-H atoms			
Protein	1540		2956
β-Me	2		
Mg	1		
Water	111		219
Average B (Å^2^)			
Chain A	20.2		42.8
Chain B			44.1
Solvent	30.0		48.8
Geometry			
rmsd bond length (Å)	0.016		0.003
rmsd bond angle (°)	1.572		0.64
PDB code	4MO7		4MXM

As shown in **Fig. 7A & 7B**, *pfi*T displays a completely α-helical structure that consists of ten α-helices. The structure of native *pfi*T in the C2 crystal form comprises residues 11–198. The N-terminal 10 residues are presumably disordered. The missing N-terminal residues can be traced without ambiguity for one monomer within the dimer of the Se-Met *pfi*T in the *P*2_1_ crystal form (**Fig. 7B**). The N-terminus forms a short α-helix (α1) together with five residues GPLGS resulted from the GST-tag vector (**Fig. 7B**).

The Se-Met *pfi*T was crystallized as a dimer, which is consistent with results from our AUC solution studies indicating that *pfi*T exits as a dimer in solution (**Fig. 5A & 5B**). Although there is only one molecule in the asymmetric unit for the C2 crystal form of native *pfi*T, a dimer very similar to that in the *P*2_1_ crystal form can be readily formed through crystallographic symmetry (**Fig. 7C**). Therefore, results from both crystal structure and solution studies indicated that *pfi*T exits as a dimer.

VAST [28] search indicated that *pfi*T belongs to the TetR-family transcription regulators [29], [30]. The structure of *pfi*T is most close to that of a putative TetR family protein from *Vibrio parahaemolyticus* (PDB code: 3HE0) (Cuff ME, Hendricks R, Moy S, Joachimiak A, unpublished data) with a root-mean-square-deviation of 1.72 Å (**Fig. 7D**). At a structural level, the TetR proteins all possess a helix-turn-helix DNA-binding domain (DBD) at their N-terminal ends, and have highly divergent C-termini postulated to be involved in the binding of inducing compounds [29], [30]. It is known that the TetR repressor function requires a TetR dimer to bind the DNA elements [31], [32], [33]. Similarly, the *pfi*T dimer is arranged in this manner of other TetR, as the dimers are all formed through the C-terminal helices [31], [32], [33], [34], [35], [36], [37] (**Fig. 7E**). However, the DBDs of the *pfi*T dimer are about 50Å apart, contrasting to an average 34 Å distance for most other TetR repressors in their active forms [31], [32], [33] (**Fig. 7E**). Similar widely-opened DBDs were also observed in the dimer of MexZ, a key repressor responsible for antibiotic resistance in *P. aeruginosa* [36]. In this regard, it has been proposed that the widely separated DBD dimer represents the released form of TetR [36].

## Discussion

Superantigens (SAgs) activate large fractions of T cells expressing particular T-cell receptor (TCR) β chains, and are the pathogenic factors of many human diseases such as toxic shock syndrome and autoimmune diseases [4]. Although much is known about the structures and functions of the pyrogenic SAgs encoded by staphylococcal and streptococcal bacteria, far less is known about the non-pyrogenic SAgs, such as the Crohn's disease (CD)-associated protein I2 (*pfi*T). I2 and its full-length protein *pfi*T encoded by *P. fluorescens* are novel enteric SAgs; they are important pathogenic factors and serological markers of CD, an inflammatory bowel disease. The discovery of pathogenic factor and serological marker I2 (*pfi*T) suggest that these proteins play a role in the pathogenesis of Crohn's disease. However, how *pfi*T performs its biological function and its role in Crohn's disease are not known.

In this study, we determined the structure and identified an immunologic function of *pfi*T. Based upon the high resolution crystal structure of *pfi*T, we found that *pfi*T belongs to the TetR transcription regulator protein family. The TetR family members are key players in multidrug resistance, virulence, and pathogenicity processes caused by bacterial pathogens. They act as repressors by binding to consecutive DNA major grooves through the N-terminal DBDs [31], [32], [33], [34], [35], [36]. TetR proteins control the expression of genes involved in multidrug resistance, enzymes implicated in catabolic pathways, biosynthesis of antibiotics, osmotic stress, and pathogenicity of gram-negative and gram-positive bacteria [29], [30]. TetR proteins function as a dimer to bind their DNA elements [31], [32], [33]. Our results demonstrated that *pfi*T exists as a dimer in solution; and crystal structure also confirmed that *pfi*T is a dimer at structural level. Structure-based alignment of sequences of *pfi*T with those of TetR members with known structures of the protein-DNA complexes indicated that most of residues important for TetR members to bind their DNA elements are conserved in the *pfi*T sequence (**Fig. 7F & 7H**). Similar to QacR and TetR, *pfi*T also displays a positively charged surface at the putative DNA binding site (**Fig. 7G**). Although it is currently unknown whether *pfi*T binds DNA, these structural evidences suggest that *pfi*T might also function as a transcription regulator, like other TetR members. However, further investigation will be required to determine whether *pfi*T acts as both as a SAg and as a DNA-binding protein.

*Pseudomonas* is a large genus of Gram-negative aerobic γ proteobacteria, belonging to the family *Pseudomonadaceae* containing 191 species [38]. The genomes of many of these species have been sequenced [39], [40], [41], [42], [43]. BLAST search [44] indicated that many putative TetR-family members of the *Pseudomonas* bacteria share high sequence homology with *pfi*T (**Fig. S1A**). In addition, putative TetR members of a number of other bacterial species also display significant sequence homology with *pfi*T (**Fig. S1B**). It is currently unknown whether these other TetR members possess SAg activity. Nonetheless, our study suggests a new function for this group of proteins, at least for *pfi*T and PA2885.

We further investigated the function of the CD-associated enteric SAg *pfi*T. Our data revealed that *pfi*T can stimulate the activation of not only murine splenocytes, as previously reported [9], [11], but also human peripheral blood T cells. We demonstrated that the class II MHC molecule is the human receptor for the SAg *pfi*T. Addition of antibodies against all three class II HLA alleles greatly reduced the binding of *pfi*T to human PBMC. Addition of anti-HLA-DR antibody alone also greatly reduced *pfi*T binding to human PBMC, whereas anti-DQ and anti-DP antibodies only moderately reduced the binding. The results indicated that HLA-DR, and to a lesser extent, HLA-DQ and HLA-DP, is responsible for *pfi*T binding. Moreover, using purified recombinant proteins, we demonstrated that *pfi*T and HLA-DR interacted directly. Competition binding data suggested that the *pfi*T-binding site on HLA-DR overlaps with that for MAM, a phylogenetically and structurally distinct SAg.

Both *pfi*T and MAM are more selective for HLA-DR [45]. Using a genetic analysis, MAM was found to be immunologically active (T cell activation) in the context of murine H-2E (the murine homologue of HLA-DR), but also certain HLA-DQ and homologue H-2A alleles [45]. Using an antibody-blocking analysis, *pfi*T also showed immunologic activation with H-2A^b^, but not H-2E or other HLA-D molecules ([9], [11], and present study). What might be the basis for this overlapping but distinct MHC-dependence of *pfi*T and MAM? Whereas staphylococcal and streptococcal SAgs share similar three dimensional structures, SAgs from other sources display various structural folds, ranging from completely β-sheets to completely α-helices [4], [24], [46]. Our structural study indicated that the structures of both *pfi*T and MAM are completely helical, although *pfi*T displays a different folding pattern [24]. Our competition binding measurements indicated that MAM can compete binding of *pfi*T to its human receptor HLA-DR. We speculate that the distinct folding patterns might yield differences in molecular contacts that could account for the divergent MHC fine specificities of these two SAgs. Alternatively, these differences may reflect the outcome of genetic versus antibody analysis. These questions await integrated assessment of these two SAgs using the same immunologic assay context, and direct structural analysis of binding with HLA-DR and H-2A.

In summary, the structural and functional studies of the *pfi*T-MHC interactions provide detailed insights into how a novel enteric SAg is recognized by its human receptor. Immune activity to I2, the peptide antigen encoded by *pfi*T, is a distinguishing biomarker for disease state and clinical phenotype of Crohn's disease [47], [48]. There is an intensifying focus on the interplay of environment and microbiota in Crohn's disease, both as a source of antigens in adaptive immune activation, and as a source of microbial products targeting innate immune and epithelial function [20], [49], [50]. This structural and functional study provides new insights into the SAg properties of *pfi*T, and accordingly, how *P. fluorescens*, a ubiquitous environmental microbial commensal, has emerged as an immunologic feature of Crohn's disease.

## Materials and Methods

### Ethics statement

Animals were housed and cared in accordance with standards of the Association for Assessment and Accreditation of Laboratory Animal Care (AAALAC) in AAALAC accredited facilities, and all animal procedures were performed according to protocols approved by the Institutional Animal Care and Use Committees of the Wadsworth Center Institutional Animal Care and Use Committee. Our institutional program that oversees the review & approval of animal use protocols uses the standards delineated in the Eighth edition of the “Guide For the Care and Use of Laboratory Animals” (the *Guide*), the “PHS Policy on Humane Care and Use of Laboratory Animals”, and the Animal Welfare Act Regulations (AWARs) for covered species. The Wadsworth Center is accredited by AAALAC International, and is an OLAW-Assured research institution (PHS Animal Welfare Assurance Number A3183-01).

The studies using anonymized human blood samples purchased from Research Blood Component was exempt and approved by the Institutional Review Board Committee of the Wadsworth Center.

### Antibodies

Primary antibodies used in this study were anti-HLA-DR G-7 (Santa Cruz Biotechnology) and L243 (ATCC), anti-HLA-DQ (IVD12) (ATCC), anti-HLA-DP (BD Biosciences), anti-HLA-DR, DQ, DP (Tu39, BD Pharmingen), anti-CD19-APC (LT19, Abcam), anti-CD45-APC-H7 (2D1, BD biosciences), anti-CD3 (HIT3a, BD Biosciences), and anti-CD3-PerCP (MEM-57, Abcam), anti-GST (GE HealthCare). Control IgG 2H11 was a gift from Dr. Ellis Reinherz of Dana-Farber Cancer Institute.

### Cloning, expression, and purification of *pfi*T, PA2885, and MAM

Full-length I2 encoded by *P. fluorescens* (*pfi*T) and its close homolog PA2885 from *P. aeruginosa* were cloned into pGEX-6P-1 expression vector (GE HealthCare) by grafting the inserts from the pQE30 vector (Qiagen). The His-tag *pfi*T and PA2885, prepared using the pQE-30 vector, were expressed and purified as described previously [51]. Expression and purification of *pfi*T, PA2885, MAM, and the selenomethionine-substituted (Se-Met) *pfi*T as GST-fusion proteins were carried out as described previously [24]. The GST-removed proteins were obtained by digestion of the GST-fusion proteins with the PreScission protease (GE HealthCare).

### Preparation of mouse spleen lymphocytes

Spleens from naive mice were harvested by grinding them with frosted slides. Harvested cells were washed with Hank's balanced salt solution (HBSS) and then taken in 0.5 ml of hypotonic ammonium chloride solution to lyse the red blood cells (RBCs). Spleen lymphocytes free of RBC were then washed twice with HBSS by centrifugation, counted with a hemocytometer, and kept in complete tumor medium at 4°C until use.

### Preparation of human PBMC

Buffy coats of human blood samples were purchased from Research Blood Component (Brighton, MA). 20 ml of Histopaque-1077 (Sigma) was pipetted into 50 ml conical centrifuge tubes and allowed sufficient time to reach the room temperature, after which 20 ml of fresh blood buffy coat was layered slowly on top of the Histopaque layer and centrifuged at 400 g for 30 minutes at room temperature. After centrifugation, the upper fluid layer was slowly removed, and the interfacial cellular layer was collected and transferred to another fresh 50 ml conical centrifuge tube. Cells were washed with phosphate buffered saline (PBS) once at room temperature and once at 4°C by centrifugation. Human T lymphocytes were further isolated from purified PBMC using the Pan T cell isolation kit II (Miltenyi Biotec Inc.) according to the manufacturer's instructions. The non-T cell components were also collected to serve as the antigen presenting cells in T-cell proliferation assay. Cells were counted by a hemocytometer, and either placed in RPMI1640 medium with 10% FBS on ice for culturing, or frozen at −80°C for future use.

For CFSE labeling experiment, purified T lymphocytes was labeled with 5(6)-carboxyfluorescein diacetate *N*-succinimidyl ester (CFSE) (Sigma), according to the manufacturer's instructions.

### Lymphocyte proliferation assay

1×10^6^ lymphocytes isolated either from mouse spleen or from human PBMC were cultured with stimulating agents at a volume of 200 µl/well in CTM or RPMI1640 medium with 10% FBS in a 96 well culture plate. After 48 hour, 0.5 µCi of [^3^H] thymidine (New England Nuclear, Boston, MA) was added to the cultures and 16 h later the cells were harvested and [^3^H]thymidine incorporation was measured using a 1205 beta plate counter (Wallac, Gaithersburg, MD).

For CSFE experiments, CFSE-labeled human T cells (2×10^6^ cells per well) were cultured with 1×10^5^ antigen-presenting cells in the presence of various SAgs or controls in a 24-well flat-bottomed plate. After 96 hours, cells in each well were washed and resuspended with phosphate buffered saline (PBS). Anti-CD3-PerCP antibody was added and incubated at room temperature in dark for 20 minutes, and then washed by 1× PBS. Cells were finally resuspended in 500 µl 1× PBS for FACS analysis using a Becton Dickinson FACSCalibur (BD Biosciences).

### ^125^I binding assay

Purified *pfi*T, PA2885, and MAM were labeled with ^125^I as described previously [22]. Cells (5×10^5^ cells/sample) were incubated with ^125^I-labeled proteins (25 nM, in 300 ml of PBS containing 1% BSA, 1 µM ZnCl_2_, and 0.02% NaN_3_). The reaction mixture was incubated at room temperature for two hours, washed with 1 ml PBS. Cells bound with labeled SAg were recovered after centrifugation over an oil cushion, and counted in a gamma counter.

### FITC-protein binding assay

Purified *pfi*T, PA2885, and MAM were labeled with FITC according to the manufacturer's instructions. Human PBMCs (5×10^5^ cells/sample) were incubated in a 50 µl volume with buffer control or various antibodies (100 nM) at room temperature for 20 minutes, then 50 µl of FITC-labeled proteins at 100 nM concentration was added along with anti-CD19-APC, and incubated in dark for 20 minutes. Cells were washed with 1× PBS, spun at 400 g for 5 minutes, and resuspended in 500 µl PBS for FACS analysis using a Becton Dickinson FACSCalibur (BD Biosciences).

### GST pull down assay

GST, GST-MAM and GST-*pfi*T (200 µg) were immobilized on glutathione-sepharose 4B beads (GE HealthCare) and aliquoted at 10 µg/tube). Raji cell lysate (15 µl) or purified recombinant HLA-DR1/HA complex was applied to the aliquoted beads, and incubated at 4°C for 1 hour. The incubated beads were washed four times with a buffer containing 20 mM Tris, pH 7.4, 0.1 mM EDTA, and 100 mM NaCl), and subjected to western blot analysis using anti-HLA-DRα (G-7, Santa Cruz) or anti-GST antibody (GE HealthCare). Binding affinity (*K_D_*) or *IC_50_* was evaluated by non-linear regression using the Prism software (GraphPad Software).

### Analytical ultracentrifugation (AUC) sedimentation velocity

Sedimentation-velocity (SV) experiments were conducted at 20°C in a Beckman Optima XL-I analytical ultracentrifuge at a rotor speed of 50,000 rpm. Double-sector cells were loaded with 400 µl of *pfi*T at 3.6 µM and with 410 µl of reference solutions, respectively. Unless otherwise specified, the reference solution is Hepes-buffered saline (HBS) containing 10 mM Hepes buffer (pH 7.4), 150 mM NaCl, 2 mM DTT. Data were recorded with absorbance detection at wavelengths of 280 nm. Absorbance profiles were analyzed with the software SEDFIT (http://www.analyticalultracentrifugation.com) [27], using a model for continuous sedimentation coefficient distributions *c*(*s*) [26]. Distributions were calculated with maximum entropy regularization at a predetermined confidence level of 1 standard deviation.

### AUC sedimentation equilibrium

Sedimentation equilibrium studies were conducted at a temperature of 20°C and at three rotor speeds for each protein or mixture. The individual protein or protein mixture (100 µl) at various concentrations in HBS was loaded into an Epon double-sector centerpiece. Reference cells were loaded with 110 µl of reference solution. For *pfi*T alone, sedimentation equilibrium of *pfi*T was analyzed at concentrations of 9 µM and 18 µM, with three rotor speeds (20,000, 25,000, and 30,000 rpm). For the *pfi*T/HLA-DR1/HA complex, mixtures of *pfi*T and DR1 at various molar ratios and concentrations, (66∶6.5, 44∶13, and 8∶24 in a real micromolar ratio of concentrations for *pfi*T:HLA-DR1), were used for sedimentation equilibrium analysis with three rotor speeds (20,000, 25,000, and 30,000 rpm).

Equilibrium absorbance profiles were acquired at either 280-nm (for *pfi*T alone) or 300-nm (for the *pfi*T-DR1 complex) wavelength. The equilibrium sedimentation data were analyzed using the software SEDPHAT (http://www.analyticalultracentrifugation.com) [27]. Data analysis was performed by global least-squares analysis of data from multiple concentrations and multiple rotor speeds, using conservation of mass constraints [27].

### Crystallization, X-ray data collection, structure determination and refinement

Purified *pfi*T was concentrated to about 10–15 mg/ml with a buffer of 10 mM Tris-HCl, pH 8.0, 100 mM NaCl, 2 mM DTT. Crystals of *pfi*T were grown at room temperature in hanging drops, by mixing 2 µl of protein solution with an equal volume of reservoir solution containing 4–8% PEG 3350, 0.1 M Tris, pH 8.0, 0.2 M ammonium acetate, 5 mM MgCl_2_, 5 mM DTT, 5% isopropanol, and 5% glycerol. Microseeding was used to produce large crystals.

Native *pfi*T crystals belong to space group C2 (**Table 1**). The Se-Met substituted *pfi*T crystals belong to space group *P*2_1_ (**Table 1**). Prior to data collection, all crystals were transferred to a reservoir solution containing 25% glycerol, and then flashed-cooled under a nitrogen stream at 100K, and stored in liquid nitrogen. Diffraction data of native crystal were collected to 1.7 Å resolution at 100K at beamline X4A of the National Synchrotron Light Source (NSLS) (Brookhaven National Laboratory). In order to solve the structure, diffraction data of the Se-Met crystals were collected to various resolutions (2.4 Å to 1.95 Å). All of the data were processed and scaled using HKL2000 [52] (**Table 1**).

The crystal structure of *pfi*T was first determined at 2.4 Å resolution using the single anomalous diffraction (SAD) phasing method with a SAD data collected at the Se peak wavelength, using the program SOLVE [53]. The electron density map was improved using the program RESOLVE [54]. Fragments containing about 55% of the *pfi*T residues could be automatically traced by the program RESOLVE. The model of a *pfi*T monomer was finally completed by manually fitting the electron density map with the *pfi*T sequence using the program TURBO FRODO [55]. The second *pfi*T molecule of the dimer in the asymmetric unit was then generated using the NCS symmetry. The structure was then fully refined, using a diffraction data at higher resolution (1.95 Å) with the PHENIX program suite [56]. The crystal structure of native *pfi*T was determined using the molecular replacement methods using PHENIX [56]. Structural refinement was carried out using PHENIX [56] (**Table 1**).

The final refinement statistics are summarized in **Table 1**. Atomic coordinates have been deposited in the Protein Data Bank as entries 4MO7 and 4MXM.

## Supporting Information

Figure S1 **Sequence alignment of** ***pfi*** **T with representative TetR-family members.** (**A**) Sequence alignment of *pfi*T with representative putative TetR members within *Pseudomonas* families. Residues are colored according to the extent of their sequence conservation: >90% conserved (red); 50–90% conservation (blue); less or not conserved (<50%) (black). (**B**) Sequence alignment of *pfi*T with representative putative TetR members of other bacterial species. Abbreviations used here include: *P., Pseudomonas; H., Hahella; B., Bermanella; M., Marinobacter; Al., Alcanivorax; A., Acinetobacter; G., Glaciecola; Ps., Pseudogulbenkiania; Ma., Marinithermus*.
(DOCX)
