# Supplementary material for: Pfit Is a Structurally Novel Crohn's Disease-Associated Superantigen
Source: PLoS Pathog. 2013 Dec 26;9(12):e1003837. doi: 10.1371/journal.ppat.1003837 (PMC3873459; doi:10.1371/journal.ppat.1003837)
Supplement: Figure S1 — Sequence alignment of pfi T with representative TetR-family members. (A) Sequence alignment of pfiT with representative putative TetR members within Pseudomonas families. Residues are colored according to the extent of their sequence conservation: >90% conserved (red); 50–90% conservation (blue); less or not conserved (<50%) (black). (B) Sequence alignment of pfiT with representative putative TetR members of other bacterial species. Abbreviations used here include: P., Pseudomonas; H., Hahella; B., Bermanella; M., Marinobacter; Al., Alcanivorax; A., Acinetobacter; G., Glaciecola; Ps., Pseudogulbenkiania; Ma., Marinithermus. (DOCX) [file ppat.1003837.s001.docx]

**A.**

**1 10 20 30 40 50 60 70 80 90 100**

**pfiT MDEHKALGVM RTMVDSGQLT DPESARGKLL QTAAHLFRNK GFERTTVRDL ASAVGIQSGS IFHHFKSKDE ILRAVMEETI HYNTAMMRAS LEEASTVRER VLALI**

**PA2885 M LELVATGQLT DPESARGKLL QTAAHLFRSK GYERTTVRDL ASAVGIQSGS IFHHFKSKDE TLRSVMEETI LYNTALMRAA LADAEDLRER VLGLI**

***P. aeruginosa PA7* MDDQKAREVM LELVASGQLT DPESARGKLL QTAAHLFRSK GYERTTVRDL ASAVGIQSGS IFHHFKSKDE ILRSVMEETI LYNTALMRAA LADAQDLRER VLGLI**

***P. citronellolis* M LELVANGQVT DPESARGKLL QTAAHLFRAK GYERTTVRDL AGAVGIQSGS IFHHFKSKDE ILRSVMEETI LYNTALMRAA LADAEDLRER LLALI**

***P. synxantha BG33R* MDEHKALGVM RAMVAGGRLT DPESARGKLL QTAAHLFRNK GFERTTVRDL ASAVGIQSGS IFHHFKSKDE ILRAVMEETI HYNTAMMRAA LEEASTVRER VLALI**

***P. sp* R81 MDEHKALRVM RTMVEAGQLT DPDSARGKLL QTAAHLFRNK GFERTTVRDL ASAVGIQSGS IFHHFKSKDE ILRAVMEETI HYNTAMMRAS LEEASNVRER VLALI**

***P. extremaustralis 14-3b* MDEHKALQVM RTLVDGGELT DPDSARGKLL QTAAHLFRNK GFERTTVRDL AGAVGIQSGS IFHHFKSKDE ILRAVMEETI RYNTALMRAS LEEASNVRER VLALI**

***P. chlororaphis O6* MDEQKALLVM RQLVANGQLT DPDSARGKLL QTAAHLFRNK GYERTTVRDL ASAVGIQSGS IFHHFKSKDD ILRAVMEETI HYNTALMRAE LAEAGSVRER VLALI**

***P. protegens Pf-5* MDEQKAQQVV RELIASGQVT DPESARGKLL QTAAHLFRNK GYERTTVRDL ASAVGIQSGS IFHHFKSKDD ILRAVMQETI HYNTALMRAE LAEAGSVRER VLALI**

***P. brassicacearum NFM421* MDEQKALGVM RELVDNGQLT DPDSARGKLL QTAAHLFRNK GFERTTVRDL ASAVGIQSGS IFHHFKSKDE ILRAVMEETI RYNTALMRAA LAEADSVRER VLALI**

***P. mandelii JR-1* MDEQKALGVM RELVDSGQLT DPDSARGKLL QVAAHLFRNK GYERTTVRDL AGAVGIQSGS IFHHFKSKDE ILRAVMEETI RYNTALMHAA LAEAGNVRER VLALI**

***P. fuscovaginae UPB0736* MNEQQAQQVM ADLVQAGQLT DPDSARGKLL QTAAHLFRNK GYERTTVRDL ASAIGIQSGS IFHHFKSKDE ILRAVMEETI LYNTALMRAS LFEAGSVRER VLALI**

***P. mendocina ymp* MDEHRAQAVM QELVASGQVT DPESARGKLL QMAAHLFRSK GYERTTVRDL AAAIGIQSGS IFHHFKSKDE ILRSVMEETI VYNTALMRAA LAEAQGTRER LLALI**

***P. pseudoalcaligenes CECT 5344* MDEHRAQAVM QELVDSGQVT DPDSARGKLL QMAAHLFRSK GYERTTVRDL AAAIGIQSGS IFHHFKSKEE ILRSVMEETI VYNTALMRAA LAEAQGTRER LLALI**

**110 120 130 140 150 160 170 180 190 200**

**pfiT RCELQ SIMGGSGEAM AVLVYEWRSL SAEGQAHVLA LRDVYEQIWL QVLGEAKAAG YIRGDVFITR RFLTGALSWT TTWFRAQGSL TLEELAEEAL LMVLKSD**

**PA2885 RCELQ SIMGGTGEAM AVLVYEWRSL SAEGQAYILG LRDIYEQMWL DVLGEARLAG YCQGDPFILR RFLTGALSWT TTWFRPEGPM SLDQLAEEAL ALVIKNAG**

***P. aeruginosa PA7* RCELQ SIMGGTGEAM AVLVYEWRSL SAEGQEYILG LRDIYEQMWL EVLGEARLAG YCQGDPFILR RFLTGALSWT TTWFRPEGPM TLDQLAEEAL ALVLKDA**

***P. citronellolis* RCELQ SIMGGTGEAM AVLVYEWRSL SLEGQAYILG LRDIYEQMWL DVLGEARAAG YCEGDPFILR RFLTGALSWT MTWFRQDGSM TLDDLAEQAL LLVYKAA**

***P. synxantha BG33R* RCELQ SIMGGSGEAM AVLVYEWRSL SAEGQAQVLA LRDVYEDIWL QVLGEAKTAG YIRGDVFITR RFLTGALSWT TTWFRTQGSL TLEELAEEAL LMVLKAD**

***P. sp* R81 RCELQ SIMGGSGEAM AVLVYEWRSL SAEGQAQVLA LRDVYEAIWL QVLGEAKDAG YIRGDVFITR RFLTGALSWT TTWFRAEGSL TLEQLAEEAL LMVLKAD**

***P. extremaustralis 14-3b* RCELQ SIMGGSGEAM AVLVYEWRSL SAEGQARVLA LRDVYEEIWL QVLGEAKVAG YIRGDVFITR RFLTGALSWT TTWFRAEGSL TLEELAEQAL LMVLKAD**

***P. chlororaphis O6* RCELQ SIMGGSGEAM AVLVYEWRSL SAQGQADVLA LRDIYEQIWL QVLGEAKEAG FIKGDVFITR RFLTGALSWT TTWFRAEGSL TLEQLAEEAL TLVLNEP**

***P. protegens Pf-5* RCELQ SIMGGSGEAM AVLVYEWRSL SPEGQAAVLA LRDIYEQIWL QVLGEAKEAG FIKGDVFITR RFLTGALSWT TTWFRAEGSM TLEQLAQEAL LLVLKDH**

***P. brassicacearum NFM421* RCELQ SIMGGTGEAM AVLVYEWRSL SQDGQRHVLA LRDIYEDLWL EVLGQAKEAG YIRGDVFITR RFLTGALSWT TTWFRAEGSL SLDELAEQAL ILVLEEKQ**

***P. mandelii JR-1* RCELQ SIMGGSGEAM AVLVYEWRSL SEDGQAQVLA LRDIYEALWL QVLGEAKDAG FIRGDVFITR RFLTGALSWT TTWFRAGGSM SLDQLADEAL ILVLEEK**

***P. fuscovaginae UPB0736* RCELQ SIMGGTGEAM AVLVYEWRSL SPQAQAKVLA LRDLYEQIWL QVLGEAREAG YIKGDVFIAR RFLTGALSWT TTWFRPQGNL TLDQLADEAL ALILKDG**

***P. mendocina ymp* RCELQ SIMGGTGEAM AVLVYEWRSL SADGQAQVLA LRDTYEQIWL AVLGEAREAG YFQGDPFIQR RFLTGALSWT TTWFRSQGPM SLDQLAEEAL SLVCKDA**

***P. pseudoalcaligenes CECT 5344* RCELQ SIMGGTGEAM AVLVYEWRSL SEEGQAQVLA LRDTYEQIWL AVLGEAREAG YFKGDPFIQR RFLTGALSWT NTWFRSQGPM TLDQLAEEAL SLVCKEA**

**B.**

**10 20 30 40 50 60 70 80 90 100 110**

**pfiT MDEHKALG -VMRTMVDSG QLTDPESARG KLLQTAAHLF RNKGFERTTV RDLASAVGIQ SGSIFHHFKS KDEILRAVME ETIHYNTAMM RASLEEASTV RERVL**

***H. chejuensis KCTC 2396* MK -QLAQLIEEG RITDPQSARG RLLAKAAHLF KEKGYERTTV RDLAASVGIQ SGSIFHHFRS KEDILFAVME ETILYITAKM REALEQAPTP RDRLL**

***B. marisrubri* MNL DALDTVDTRD PVLKDLIHKG QVTDPHSAKG KLMRAAAHLF KSKGYERTTV RELGAAVGIQ SGSLFHHFKS KEEILLAVME ETIIINMARM NAALSDSKDA GEKLH**

***M. algicola DG893* MNQD SILRSLVADN LVSDPAGARG RLLHEAAKLF RDKGYERTTV RDLAAAVGIQ SGSLFHHFRT KEEILKAVMV ETIRLNTALM QAAVDAASTH REKLQ**

***M. manganoxydans MnI7-9* MFQVELKASY ANRVAVVSQN QILQSLIAEN LVSDPEGARG RLLTEAARLF REKGYERTTV RDLAAAVGIQ SGSLFHHFRT KEEILKAVMV ETIRLNTALM QAALEAADTS RQKLR**

***M. adhaerens HP15* MFQVELKASY ANRVAVVSQN QILQSLIAEN LVSDPKGARG RLLTEAARLF REKGYERTTV RDLAAAVGIQ SGSLFHHFRT KEEILKAVMV ETIRLNTALM QAALEAADTS RQKLR**

***M. aquaeolei VT8* MAFVNQN PILQSLIAEQ LVSDPSSARG RLLNEAARLF RDKGYERTTV RDLAAAVGIQ SGSLFHHFRT KEEILKAVMV ETIRLNTALM QAAADQADSA REKLR**

***M. hydrocarbonoclasticus ATCC 49840* MAFVNQN PILQSLIAEQ LVSDPASARG RLLNEAARLF RDKGYERTTV RDLAAAVGIQ SGSLFHHFRT KEEILKAVMV ETIRLNTALM QAAADQADSA REKLR**

***A. hongdengensis A-11-3* MVESLAIN GIDD--SPRG RLLSAAAHLF RDKGFDRTTV RDIAAAVGIQ SGSIFHHFKS KEDILYAVME EVIHFNTERL RQAIASQDSA RDKLR**

***A. sp. DG881* MAESLAIN GIDD--SPRG RLLSAAAHLF RDKGFDRTTV RDIAASVGIQ SGSIFHHFKT KEDILYAVME EVIHFNTERL RQAVASHATG KEQLR**

***A. borkumensis SK2* MAGLMAIH GIDD--SPRG RLLSAAAHLF RDKGFDRTTV RDIAASVGIQ SGSIFHHFKT KEDILYAVME EVIHFNTERL RQAVVAHSTG QEQLR**

***A. dieselolei B5* MTQARAVH GIDD--SPRG RLLSAAAQLF RDKGYERTTV RDIAAVVGIQ SGSIFHHFKT KEEILFAVME EVIHFNTERL RAAIAAENRP VERLR**

***A. baumannii OIFC021* MI ATSIEQIPNI SCFD-DSPRG RLLRGAAYLF HKQGYDKTTV RELAQFIGIQ SGSLFHHFKS KDDILAHVME ETIIYNLARL EEAAHQSTDP EQQLR**

***A. oleivorans DR1* MI ATSIEQIPNI SCFD-DSPRG RLLRGAAYLF HKQGYDKTTV RELAQFIGIQ SGSLFHHFKS KDDILAHVME ETIIYNLARL QDAAAQSTDP EQQLR**

***A. nosocomialis Ab22222* MI ATSIEQIPNI SCFD-DSPRG RLLRGAAYLF HKQGYDKTTV RELAQFIGIQ SGSLFHHFKS KDDILAHVME ETIIYNLARL EEAAHQSTDP EQQLR**

***A. calcoaceticus RUH2202* MI ATSIEQIPNI SCFD-NSPRG RLLQGAAYLF HKQGYDKTTV RELAQFIGIQ SGSLFHHFKS KDDILAHVME QTIIYNLARL EDAANQSTDP EQQLR**

***A. haemolyticus ATCC 19194* MI ATSIEQIPKI VCFD-DSPRG RLLLGAAYLF YKQGYDKTTV RQLGEFIGIQ SGSLFHHFKS KDDILATVME QTIIYNFARL KEAAERSNDP EQQLR**

***A. junii SH205* MLQYTL IKRDRRTGMI ATSIEQIPKI VCFD-DSPRG RLLLGAAYLF YKQGYDKTTV RQLGEFIGIQ SGSLFHHFKS KDDILATVME QTIIYNFARL KEAAERSNDP EQQLR**

***A. bereziniae LMG 1003* MI ANSINDIQPI ACFD-DSPRG RLLLGAAYLF HKQGYAKTTV RELANFIGIQ SGSLFHHFKS KDDILAHVMQ QTIIYNHARL LDAIKISDDP EQQLK**

***Ps. ferrooxidans 2002* MTTLEKN TPLEENSRRM ELVRAAAKLF RDQGYERTTV RDLGNAVGLQ SGSLFYHFRT KEEILVAVMA LGISSTTEQL ERAIAKAGST REKLS**

***Ma. hydrothermalis DSM 14884* MHPTPRGRRA GILAAAARLF RTQGFERTTV RQIADAVGLQ SGSLFHHFKS KEAILLAVME DGVRQAIAAA DRALAAADTP PERLR**

***G. arctica BSs20135* MLIPQLVSEG LLTEPNSAKG RLLAASAALF KQKGFSRTTV RDIAAEVGIL SGSIFHHFAN KESILRTIML EAIYLVLARM KLTIADLDSC EDKMR**

**120 130 140 150 160 170 180 190 200 210 220**

**pfiT ALIRC ELQSIMGGSG EAMAVLVYEW RSLSAEGQAH VLALRDVYEQ IWLQVLGEAK AAGYIRGDVF ITRRFLTGAL SWTTTWFRAQ -GSLTLEELA EEALLMVLKS D**

***H. chejuensis KCTC 2396* ALLRC ELESVLGGTG EAMTVLVYEW RSLSEPRQDE ILKLRDQYEG LWLDTLSEAR DAGLVKGDVA VLRRFLTGAL SWTITWYKPE -GSMTVEELA QQALYLVVKE**

***B. marisrubri* ALIRC ELDSVHTDTG EAMSVLVYEW RSLSPDKQKY VLQLRDEYET LWLNTIEECK QQGLIQHDAF ILRRLLTGAI GWTTTWYRPD -GNLSLEDLA KQTLLLAIK**

***M. algicola DG893* ALIRA ELESINGQTG EAMAVLVFEW RSLSEESQVY VLELRDIYEQ LWLDVLETLR QDGVLAADPF VVRRMLTGAL SWTVTWYRPD -GGLTLDDLT AQVVAMMGLT SL**

***M. manganoxydans MnI7-9* GLIRA ELESINGQTG EAMAVLVFEW RSLSAPSQGE VLELRDIYEK LWLDVLESLK REGALEADPF VVRRMLTGAL SWTVTWYKPE RGGLTLDGLT DQVMAMMAL**

***M. adhaerens HP15* GLIRA ELESINGQTG EAMAVLVFEW RSLSAPSQGE VLELRDIYEK LWLDVLESLK REGALEADPF VVRRMLTGAL SWTVTWYKPE RGGLTLDGLT DQVMAMMAL**

***M. aquaeolei VT8* ALIRA ELESINGQTG EAMAVLVYEW RSLSESSQAE VLELRDIYEN LWLSTLQQLS KEHQLGADPF ITRRMLTGAL SWTVTWYRPE RGGLTLDGLT DQVMAMLGLA QC**

***M. hydrocarbonoclasticus ATCC 49840* ALIRA ELESINGQTG EAMAVLVYEW RSLSEGSQAE VLELRDIYED LWLSTLQQLS KENQLGADPF ITRRMLTGAL SWTVTWYRPE RGGLTLDGLT DQVMAMLGLA QC**

***Al. hongdengensis A-11-3* ALLRA ELQSIVGDTA EAMAVLVTEW RCLSDDKQRK ALALRGIYEQ LWLQVLESLH REGAFRADPF IIRRLLTGMT GWVPNWFDRD -GSLSLDDLA DIMVERVVGE S**

***Al. sp. DG881* ALIRA ELQSIVGDTA EAMTVMVTEW RCLSADKQAR ALKLRGIYEQ LWLDVLSELH DQGAFTTDPF IIRRLLTGMT GWAPNWFDRE -GPLSLDDLA DIMLARVIGE SAP**

***Al. borkumensis SK2* ALIRA ELQSIVGDTA EAMTVMVTEW RCLSEDKQAR ALKLRGIYEQ LWLDVLTRLH KEGVFTTDPF IMRRLLTGMT GWAPNWFDRE -GPLSLDDLA DIILARVIGE AER**

***Al. dieselolei B5* ALVRA ELTAIVGDTS EAMTVLVTEW RCLNTDKQRE ALALRDIYEQ LWLDVLADLH KEGRFRADPF IMRRLITGMT GWTHNWFDQS -RRLSVEGLA DLIVDRVVGE GESEEGAA**

***A. baumannii OIFC021* ALIKA ELISITGDTG AAMAVLVYEW FALSKEKQNY LLKMRNEYEQ IWLDVIENLR TQGKVKHDAF IWRRLIGGAI SWTVTWYKSE -GKVKIDELT EMVLVMALK**

***A. oleivorans DR1* ALIKA ELISITGDTG AAMAVLVYEW FALSKEKQDD LLKMRNEYEQ IWLDVIEKLR TEGKVKHDAF IWRRLVGGAI SWTVTWYKAE -GKVKIDELT EMVWEMALK**

***A. nosocomialis Ab22222* ALIKA ELISITGDTG AAMAVLVYEW FALSKEKQDY LLKMRNEYEQ IWLDVIEKLR TEGKVKHDAF IWRRLVGGAI SWTVIWYKAE -GKVKIDELT EMVWEMALK**

***A. calcoaceticus RUH2202* ALIKA ELISITGDTG AAMAVLVYEW FALSKEKQDY LLKMRNEYEQ IWLDIIEKLR TQGKIKHDAF IWRRLVGGAI SWTVTWYKSE -GKVKIDEVT EMVWEMALK**

***A. haemolyticus ATCC 19194* DLIRA ELISITGDTG AAMAVLVHEW FALSKEKQDY LLKMRNEYEQ VWFDVIEKLR ALGKVKHDAF IWRRLVGGSI SWTVTWYRPE -GKVKIDELT EMVWEMALK**

***A. junii SH205* DLIRA ELISITGDTG AAMAVLVHEW FALSKEKQEY LLKMRNEYEQ VWLDVIEKLR TQGKVKHDAF IWRRLVGGSI SWTVTWYRPE -GKVKIDELT EMVWEMALK**

***A. bereziniae LMG 1003* HLIKA ELISISGDTG SAMAVLVYEW FALSKPRQDE LLKLRNAYED IWLTVIEKLR ELNKVQHDAF IWRRLLGGAI AWTVTWYKPN -GKMTMDELT ETVLSMALK**

***Ps. ferrooxidans 2002* ALFHV HLNSLLGDNQ AALEVMLYEW RSVSEAAKPG LILLRDRYEA LWQAVLDEAA AAGLIKPDTR LLRRTLLGSL HWSVQWYRKD -GELSVDKLA DRMLDLVLIEQ SK**

***Ma. hydrothermalis DSM 14884* ALVRA HLETLLGPAK DALAVLLYEW RALSPEARER LIALRDAYEA RWQQVLDELA REGRAPQDTR LYRRYLLGAL NWAHEWYRPK -GELSVAVLA ERFAAFALGEA PKGGRDGV**

***G. arctica BSs20135* ALLRC ELEAIHGLNG IGFTLLSAEW RFLSDESQSE ILKLRGEYEA FHRNIYTEAK SLGHINVEPF FIRHFVRGAL IETANWYHLH -GDLPLEGLV EQIYLTTAVKW**

**Figure S1. Sequence alignment of *pfi*T with representative TetR-family members.** **(A)** Sequence alignment of *pfi*T with representative putative TetR members within *Pseudomonas* families. Residues are colored according to the extent of their sequence conservation: >90% conserved (red); 50-90% conservation (blue); less or not conserved (<50%) (black). **(B)** Sequence alignment of *pfi*T with representative putative TetR members of other bacterial species. Abbreviations used here include: *P., Pseudomonas; H., Hahella; B., Bermanella; M., Marinobacter; Al., Alcanivorax; A., Acinetobacter; G., Glaciecola; Ps., Pseudogulbenkiania; Ma., Marinithermus.*
